# RCAN1 Knockdown Reverts Defects in the Number of Calcium-Induced Exocytotic Events in a Cellular Model of Down Syndrome

**DOI:** 10.3389/fncel.2018.00189

**Published:** 2018-07-06

**Authors:** Jacqueline Vásquez-Navarrete, Agustín D. Martínez, Stéphane Ory, Ximena Baéz-Matus, Arlek M. González-Jamett, Sebastián Brauchi, Pablo Caviedes, Ana M. Cárdenas

**Affiliations:** ^1^Centro Interdisciplinario de Neurociencia de Valparaíso, Facultad de Ciencias, Universidad de Valparaíso, Valparaíso, Chile; ^2^Centre National de la Recherche Scientifique (CNRS UPR 3212), Institut des Neurosciences Cellulaires et Intégratives (INCI), Strasbourg, France; ^3^Department of Physiology, Faculty of Medicine, Universidad Austral de Chile, Valdivia, Chile; ^4^Programa de Farmacología Molecular y Clínica, ICBM, Facultad de Medicina, Universidad de Chile, Santiago, Chile; ^5^Centro de Biotecnología y Bioingeniería (CeBiB), Departamento de Ingeniería Química, Biotecnología y Materiales, Facultad de Ciencias Físicas y Matemáticas, Universidad de Chile, Santiago, Chile

**Keywords:** down syndrome, exocytosis, cholinergic vesicles, RCAN1, trisomy 16, total internal reflection fluorescence microscopy, vesicular acetylcholine transporter, pHluorin

## Abstract

In humans, Down Syndrome (DS) is a condition caused by partial or full trisomy of chromosome 21. Genes present in the DS critical region can result in excess gene dosage, which at least partially can account for DS phenotype. Although regulator of calcineurin 1 (RCAN1) belongs to this region and its ectopic overexpression in neurons impairs transmitter release, synaptic plasticity, learning and memory, the relative contribution of RCAN1 in a context of DS has yet to be clarified. In the present work, we utilized an *in vitro* model of DS, the CTb neuronal cell line derived from the brain cortex of a trisomy 16 (Ts16) fetal mouse, which reportedly exhibits acetylcholine release impairments compared to CNh cells (a neuronal cell line established from a normal littermate). We analyzed single exocytotic events by using total internal reflection fluorescence microscopy (TIRFM) and the vesicular acetylcholine transporter fused to the pH-sensitive green fluorescent protein (VAChT-pHluorin) as a reporter. Our analyses showed that, compared with control CNh cells, the trisomic CTb cells overexpress RCAN1, and they display a reduced number of Ca^2+^-induced exocytotic events. Remarkably, RCAN1 knockdown increases the extent of exocytosis at levels comparable to those of CNh cells. These results support a critical contribution of RCAN1 to the exocytosis process in the trisomic condition.

## Introduction

Down Syndrome (DS), a condition caused by the presence of an extra copy of chromosome 21, is manifested by multiple abnormalities, the most prominent features being neurological and cognitive disabilities. Although cognitive impairments vary in DS individuals from mild to moderate, working memory, language and comprehension are the most greatly impaired functions (Lanfranchi et al., 2004, 2010; Roberts et al., 2007). These cognitive deficits appear to correlate with altered brain development and morphology, particularly in the hippocampus and orbitofrontal cortex regions (Menghini et al., 2011; Carducci et al., 2013). However, neurochemical dysfunctions also seem to underlie the condition. Indeed, reduced levels of some neurotransmitters, such as glutamate, GABA, dopamine and serotonin have been found in brains of DS individuals (Godridge et al., 1987; Reynolds and Warner, 1988; Risser et al., 1997). These alterations in neurotransmission could in turn impair synaptic plasticity (Siarey et al., 1997, 1999, 2005; Kleschevnikov et al., 2004; Belichenko et al., 2007), and consequently hinder learning and memory (Morice et al., 2008).

Among the many genes overexpressed in the DS condition is Down SD critical region 1 (DSCR1), also known as Regulator of Calcineurin 1 (from RCAN1). This gene is located in the DS critical region (Fuentes et al., 1995) and encodes a protein that inhibits the Ca^2+^-dependent phosphatase calcineurin (Fuentes et al., 2000; Rothermel et al., 2000). The Rcan1 protein is highly expressed in human fetal brains (Fuentes et al., 1997), and its mRNA levels are reportedly 2–3-fold higher in post-mortem brains from Alzheimer’s disease patients and DS patients suffering from Alzheimer’s symptoms (Ermak et al., 2001). Mice models overexpressing Rcan1 exhibit impairments in long-term synaptic potentiation, learning and memory (Dierssen et al., 2011; Xing et al., 2013; Wong et al., 2015). Furthermore, Rcan1 overexpression in adrenal chromaffin cells disturbs transmitter release (Keating et al., 2008; Zanin et al., 2013). Hence, the overexpression of this protein could greatly contribute to the reduced neurotransmitter levels observed in the DS condition. However, the contribution of Rcan1 in a context where other genes of the DS critical region are also overexpressed has yet to be determined, which is most relevant to the trisomic condition.

The CTb cell line, derived from the brain cortex of trisomy 16 (Ts16) mice, an animal model of DS, overexpresses most of the genes of the DS critical region (Reeves et al., 1986), including Rcan1 (Lange et al., 2004). Similar to primary culture of central neurons from Ts16 mouse fetus, CTb cells display altered Ca^2+^ currents and cytosolic Ca^2+^ signals (Cárdenas et al., 1999; Rojas et al., 2008; Acuña et al., 2012). Cholinergic function is also impaired, which is characterized by a reduced fractional acetylcholine release (Fiedler et al., 1994; Allen et al., 2000). This latter condition could be the consequence of an altered exocytosis mechanism. To study this possibility, and the role of Rcan1 in this dysfunction, we monitored exocytosis in CTb cells expressing the vesicular acetylcholine transporter fused to the pH-sensitive green fluorescent protein (VAChT-pHluorin) by total internal reflection fluorescence microscopy (TIRFM). We also evaluated the expression of Rcan1 in CTb cells and reduced its levels to those of the CNh cell line (“*knockdown*”) via transfection with specific siRNAs. We found that CTb cells overexpress Rcan1 and exhibit a reduced number of Ca^2+^-induced exocytosis events, compared to CNh cells, a cell line established from a normal littermate. Rcan1 knockdown in the trisomic CTb cells restored the extent of exocytosis to levels comparable to those of the control cell line. This study is an important step in the quest for understanding the mechanisms contributing to neurotransmission dysfunction in the DS condition.

## Materials and Methods

### Reagents

Bafilomycin A1 (Sigma-Aldrich, St. Louis, MO, USA), bovine serum albumin (Sigma-Aldrich, St. Louis, MO, USA); Dulbecco’s modified F-12 medium (DMEM/F12; Gibco BRL, Gaithersburg, MD, USA); fetal bovine serum (Gibco BRL, Gaithersburg, MD, USA); gentamicin (Gibco/Life Technology, China); HEPES (Calbiochem, La Jolla, CA, USA); Lipofectamine 2000 (Invitrogen, Carlsbad, CA, USA); penicillin (OPKO, Chile), polyclonal antibody against Rcan1 (Santa Cruz Biotechnology); ionomycin (Calbiochem, La Jolla, CA, USA); β-tubulin antibody (Cytoskeleton, St. Denver, CO, USA); nicotine (Sigma-Aldrich, St. Louis, MO, USA); secondary antibody sheep HRP (R & D Systems, Minneapolis, MN, USA); secondary antibody donkey anti-rabbit HRP (Jackson ImmunoResearch, West Grove, PA, USA). VAChT-pHluorin was constructed as previously described (Brauchi et al., 2008). The siRNA-1 against mouse RCAN1 (sc-45481) and control scrambled siRNA (sc37007) were purchased from Santa Cruz Biotechnology Inc. (Santa Cruz, CA, USA). The siRNA-2 against mouse RCAN1 (ID: MSS285509, Catalog # 1320001) was purchased from Thermo Fisher (Carlsbad, CA, USA).

### Culture of Cell Lines and Transfection

The establishment and characterization of both the CNh and CTb cell lines have been previously reported (Cárdenas et al., 1999). Both cell lines were cultured in a 1:1 mixture of DMEM/F12 supplemented with 10% of fetal bovine serum, 50 U/ml penicillin and 100 μg/ml gentamicin at a density of 3 × 10^5^ cells/ml in 25 mm glass coverslips and incubated at 37°C in a 5% CO_2_ atmosphere until experimentation.

For cell transfections, 2 μg of VAChT-pHluorin or 1 μg of control non-targeting (NT) siRNA or siRNA against Rcan1 were incorporated into the cells using 8 μL of Lipofectamine 2000 in 42 μL of DMEM/F12 media without fetal bovine serum or antibiotics. This mixture was incubated for 15 min, and later mixed with 50 μL of DMEM/F12 for 4.5 h at 37°C in a 5% CO_2_ atmosphere. Subsequently, transfections were stopped by addition of 1 ml of DMEM/F12 supplemented with fetal bovine serum and antibiotics and kept at 37°C in a 5% CO_2_ atmosphere for 24 h.

### Determination of Rcan1 Protein Levels

Rcan1 expression was determined in non-transfected CNh and CTb cells, and in siRNA transfected CTb cells. Cells were lysed in a non-denaturing lysis buffer composed of: 300 mM NaCl, 5 mM EDTA, 50 mM TRIS HCl, 1% Triton X-100 and supplemented with 1 μM phenylmethyl sulfonylflouride, 0.1 mM leupeptine, 50 mM NaF and 0.2 mM Na_3_VO_4_. Total protein content was determined using the Quant-it Protein Assay Kit (Invitrogen, Carlsbad, CA, USA). Total proteins (100 μg) were separated by SDS-PAGE on 10% polyacrylamide gels and electrophoretically transferred to PVDF membranes (GE Healthcare Life Sciences, Piscataway, NJ, USA). Blots were preincubated with phosphate-buffered saline containing 5% bovine serum albumin and 1% Tween-20 for 2 h at room temperature. Then, the membranes were cut at approximately 40 kDa, so that Rcan1 and β-tubulin (loading control) could be probed in parallel. Afterwards, membranes were incubated overnight at 4°C with polyclonal antibodies against Rcan1 (1:500) or β-tubulin (1:1000). After primary antibody incubation and washing, incubation with a secondary donkey anti-rabbit HRP antibody (1:5000) or with an anti-sheep HRP antibody (1:5000) was performed for 1 h and detection was carried out using ECL Select Western Blotting Detection Reagent (GE Healthcare Bio-Sciences Corp., Piscataway, NJ, USA). Immunoreactive bands were detected using the image acquisition system Epichemi^3^ Darkroom. The image analysis software ImageJ 1.43 m (NIH, Bethesda, MD, USA) was used for quantification.

### Live-Cell Fluorescence Imaging

Cells were imaged using an inverted microscope (Eclipse Ti-E, Nikon, Tokyo, Japan) implemented with a 60× /1.49NA Plan APO TIRF objective (Nikon, Tokyo, Japan) and a Perfect Focus Unit TI-ND6-PFS (Nikon, Tokyo, Japan). Samples were illuminated by 488 nm laser (488-20LS, OBIS, Coherent, Santa Clara, CA, USA). Images were acquired by using a Digital Camera C11440 (ORCA-FLASH 2.0; Hamamatsu Photonics, Hamamatsu City, Japan) and the NIS-Element viewer 4.3 software (Nikon, Tokyo, Japan). Images were acquired at 300 ms intervals in stream mode.

During recordings, cells were perfused with a Krebs-Hepes solution (mM: 140 NaCl, 5.9 KCl, 1.2 MgCl_2_, 2 CaCl_2_, 10 D-glucose, 10 Hepes-NaOH, pH 7.4) and exocytosis was induced with 100 μM nicotine or 20 μM ionomycin in the perfusion solution. The extracellular Ca^2+^ concentration in the experiments with ionomycin was 4 mM. To quench extracellular VAChT-pHluorin, an acid solution containing (in mM): NaCl (140), KCl (2.4), CaCl_2_ (2), MgCl_2_ (2), glucose (10), HEPES (10), citric acid (10), adjusted to pH 7.2, 6.0 or 5.5 was used. For experiments with 100 mM HEPES, the concentration of NaCl was adjusted to keep osmolarity constant. Each recording lasted 3 min. All the experiments were performed at room temperature (20 ± 2°C).

Image sequences were analyzed with the ImageJ software (NIH, Bethesda, MD, USA) implemented with a macro to automatize spot analysis. Briefly, exocytosis events were manually pointed out in the videos, and two circular regions of interest (ROI), each one greater than 2-pixel diameter (1 pixel = 240 nm), were drawn around the selected event. One of the ROI corresponded to an exocytotic event and the other to background. Only abrupt increases in fluorescence intensity that did not shift in X, Y planes or overlap with other fluorescence events were analyzed. Fluorescence intensity of each selected ROI was measured and background was subtracted to obtain the fluorescence profile. A secondary bright spot next to the fusion event was used as intrinsic reference. The decay phase was plotted and fitted to a single exponential decay function using the software Origin 8.0 (OriginLab, Northampton, MA, USA).

### Data Analysis

For each condition, we analyzed 9–30 independent cells from at least three different cultures. Statistical significance was determined utilizing analyses of variance (ANOVA), followed by Tukey-Kramer Multiple Comparisons Test as *post hoc*. Given that time constants (τ) were non-parametrically distributed, data were statistically analyzed using Kruskal-Wallis test, followed by Dunn’s Multiple Comparisons test as *post hoc*.

### Ethics Statement

The investigators declare to know the Manual of Biosafety Regulations stipulated by CONICYT (Chile), version 2008, CDC (USA) Biosafety Manual 4th Edition, and Laboratory Biosafety, WHO, Geneva, 2005; mainly in reference to experiments with recombinant DNA and RNA and the manipulation of cell lines. This research was approved by the Biosafety Unit of the Faculty of Medicine, Universidad de Chile.

## Results

### Characterization of Exocytosis in CNh and CTb Cells

To characterize the frequency of exocytosis and kinetics of events in CNh and CTb cells, we used the VAChT-pHluorin chimera as a reporter. In this construct, pHluorin is fused to the putative first intraluminal loop of VAChT which is targeted to the acidic lumen of cholinergic vesicles. In vesicles, pHluorin is quenched and its fluorescence drastically increased when it is exposed to neutral extracellular medium upon exocytosis (Brauchi et al., 2008). In order to ascertain whether overexpressed VAChT is correctly targeted to cholinergic vesicles, we immuno-stained cells for the endogenous VAChT and evaluated colocalization by calculating the Pearson correlation coefficient (PCC). We found that VAChT-pHluorin significantly colocalized with VAChT in both CNh and CTb cells with PCC values of 0.8 ± 0.05 in CNh cells and 0.9 ± 0.03 in CTb cells (see Supplementary Figure S1).

**Figure 1:**
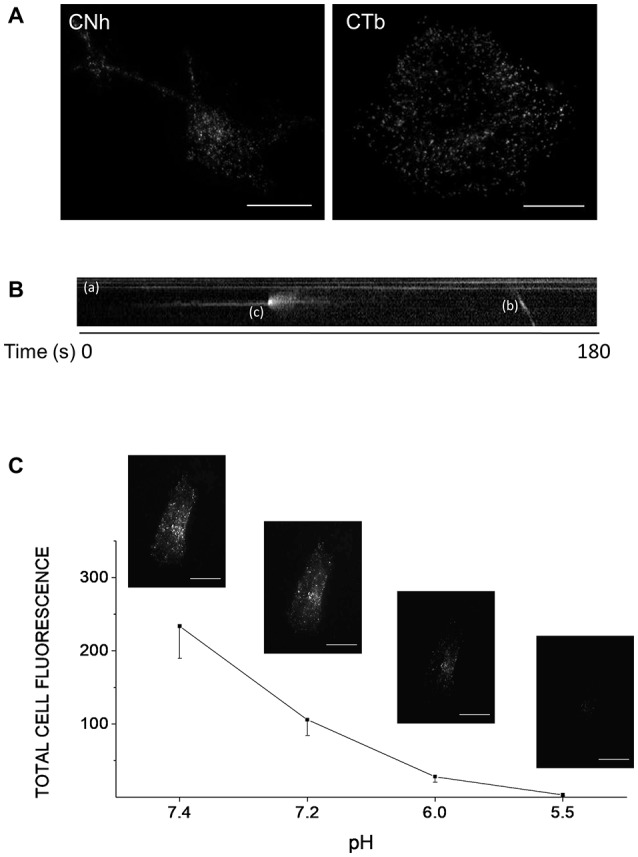
Different types of VAChT-pHluorin fluorescent spots in CNh and CTb cells. Cells cultured in 25 mm coverslips were transfected with the VAChT-pHluorin expression vector using lipofectamine 2000. Cells were visualized by TIRF microscopy 24 h later. **(A)** Expression and distribution of VAChT-pHluorin in CNh and CTb cells. Scale bar = 10 μm. **(B)** The kymograph shows three different types of fluorescent spots: **(a)** immobile spots showing constant fluorescent intensity; (b) a fluorescent spot moving along x, y positions, and **(c)** a spot that abruptly appeared and displayed a fast and transient increase in fluorescence intensity. **(C)** CNh cells were incubated in an acid solution containing (in mM): NaCl (140), KCl (2.4), CaCl_2_ (2), MgCl_2_ (2), glucose (10), HEPES (10), citric acid (10), adjusted to pH 7.4, 7.2, 6.0 or 5.5 and total fluorescence intensity was measured. Data are means ± SEM of the total cell fluorescence in arbitrary units at each pH (*n* = 7). Insets: examples of images acquired at each pH. Scale bar = 10 μm.

To monitor exocytosis and gain insight into the kinetics of single exocytotic events, we imaged cells using TIRFM. We first analyzed CNh and CTb cells maintained in resting conditions. Figure 1A shows representative TIRF images of CNh and CTb cells expressing VAChT-pHluorin. Vesicle-like structures were detected as fluorescent spots distributed along the cell body. As observed in neurons with the use of other pHluorin constructs (Atluri and Ryan, 2006), we identified different pools of fluorescent spots in CNh or CTb cells: (1) Figure 1B shows a pool of immobile fluorescent spots that exhibited constant fluorescent intensity (see spots (a) in Figure 1B). These were quenched after cell incubation with an acid solution (Figure 1C) suggesting that they corresponded to surface expression of VAChT-pHluorin. This has been previously observed with other pHluorin constructs in hippocampal neurons (Sankaranarayanan et al., 2000; Gandhi and Stevens, 2003; Fernández-Alfonso et al., 2006). (2) A small fraction of immobile fluorescent spots (app. 4%) that showed constant fluorescent intensity and it could not be quenched by an acid solution (Figure 1C). (3) Few spots that moved along x, y positions (see spot (b) in Figure 1B). Pools (2) and (3) might correspond to internalized vesicles that have not yet been reacidified, as observed in hippocampal neurons (Atluri and Ryan, 2006). (4) A fraction of spots appeared abruptly, displaying a fast and transient increase in fluorescence intensity (see spot (c) in Figure 1B); these latter types are reminiscent of exocytotic events (Gandhi and Stevens, 2003) and were further considered for analysis.

### Spontaneous Exocytosis Is Not Altered in CTb Cells

**Figure 2:**
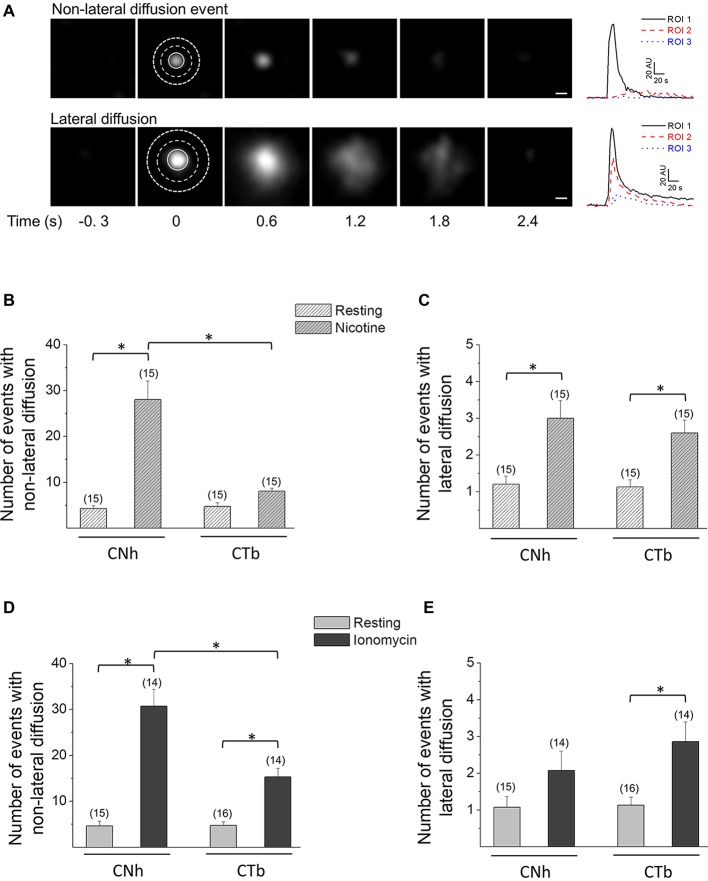
CNh and CTb cells display two types of exocytotic events. Exocytosis was visualized using TIRF microscopy in VAChT-pHluorin expressing CNh or CTb cells in resting conditions or stimulated with 100 μM nicotine or 20 μM ionomycin. **(A)** Left panels: sequence of video frames of single spontaneous fluorescence events in resting CNh cells. The upper panel shows an event with non-lateral diffusion; the bottom panel shows an event that spreads laterally. The numbers on the bottom indicate time (in seconds) relative to the onset of the exocytotic event. Scale bar = 400 nm. Right panels: fluorescence intensity profiles inside the ROI 1 (solid black line) and in the surrounding area (ROI 2 and ROI 3; dash red and dot blue lines, respectively). **(B–E)** Data are means ± SEM of the number per cell of non-lateral diffusion **(B–D)** or lateral diffusion **(C–E)** events during a 3 min recording period in CNh and CTb cells in resting conditions or stimulated with nicotine **(B–C)** or ionomycin **(D,E)**. Cells in resting conditions contain the vehicle (0.002% ethanol in **B,C** and 1% DMSO in **D,E**). Numbers in parentheses indicate the number of cells analyzed from at least three independent cultures. *p < 0.05 (ANOVA followed by Tukey-Kramer Multiple Comparisons Test).

To characterize exocytotic patterns and to determine whether exocytotic parameters were modified between CNh and CTb cells, a ROI around the exocytotic spot was delineated and changes in fluorescence intensities were measured inside and outside the ROI (see Figure 2A). With this strategy, we identified two types of VAChT-pHluorin fluorescence behavior in both CNh and CTb cells. The first was classified as non-lateral diffusion events, since VAChT-pHluorin fluorescence increased and vanished without diffusing outside the ROI (see upper panels in Figure 2A). The second, instead, showed a fast increase in VAChT-pHluorin fluorescence, which spread outside the ROI (see lower panels in Figure 2A). These latter events were termed lateral diffusion events. According to this classification, 82 ± 5% of events in CNh cells (*n* = 30) and 90 ± 3% of events in CTb cells (*n* = 29) displayed non-lateral diffusion at resting conditions, whereas less than 20% of the total events in both types of cells diffused laterally. During the 3 min recording period, exocytosis events with non-lateral diffusion amounted to 5.2 ± 0.8 in resting CNh cells (*n* = 30) and 6.2 ± 0.9 in CTb cells (*n* = 29; Table 1), whereas the events with lateral diffusion amounted to 0.9 ± 0.2 (*n* = 30) and 0.9 ± 0.3 (*n* = 29), in resting CNh and CTb cells, respectively (Table 1). No significant difference was found in the number of exocytotic events of CNh and CTb cells.

**Table 1:** Effects of high HEPES concentration and bafilomycin A1 on the fluorescence decay of non-lateral and lateral diffusion events.

	τ (s)	Number of events	Number of cells
**Non-lateral diffusion events**			
**CNh cells**			
Resting	1.4 ± 0.2	5.2 ± 0.8	30
Resting + HEPES	2.8 ± 0.3*	7.7 ± 0.7	32
Resting + bafilomycin A1	3.2 ± 0.3*	7.9 ± 0.9	14
**CTb cells**			
Resting	1.1 ± 0.1	6.2 ± 0.9	29
Resting + HEPES	4.1 ± 0.7^†^	9.2 ± 1.7	21
Resting + bafilomycin A1	3.4 ± 0.2^†^	7.0 ± 0.9	16
**Lateral diffusion events**			
**CNh cells**			
Resting	1.6 ± 0.2	0.9 ± 0.2	30
Resting + HEPES	1.2 ± 0.1	1.1 ± 0.1	30
Resting + bafilomycin A1	1.9 ± 0.1	1.3 ± 0.2	14
Lateral diffusion events			
**CTb cells**			
Resting	1.3 ± 0.2	0.9 ± 0.3	29
Resting + HEPES	1.1 ± 0.1	1.4 ± 0.2	21
Resting + bafilomycin A1	1.7 ± 0.1	1.3 ± 0.2	16

Decay time (τ) and number of exocytotic events per cell in CNh and CTb cells in resting conditions, in the absence or presence of 100 mM HEPES or 100 nM bafilomycin A1. Data are means ± SEM. *p < 0.05 compared with CNh cells in resting condition, ^†^*p* < 0.05 compared with CTb cells in resting condition (ANOVA followed by Tukey-Kramer Multiple Comparisons Test for the number of events; Kruskal-Wallis test, followed by Dunn’s Multiple Comparisons test as *post hoc* for decay times).

### Ca^2+^-Induced Exocytosis Is Reduced in CTb Cells

The release of neurotransmitters relies mostly on Ca^2+^-regulated exocytosis. Therefore, we induced exocytosis with nicotine, an agonist that reportedly evoked intracellular Ca^2+^ increases and ^3^H-choline release in CNh and CTb cells (Cárdenas et al., 1999, 2017; Allen et al., 2000; Opazo et al., 2006; Rojas et al., 2008). In CNh cells, nicotine induced 28 ± 4.1 events with non-lateral diffusion and 3 ± 0.5 events with lateral diffusion (*n* = 15), being both significantly larger than those observed in resting conditions (Figures 2B,C). In CTb cells, nicotine did not increase the amount of non-lateral diffusion events, as compared with the resting condition (Figure 2B), although this was the predominant mode of exocytosis (76 ± 2% of the events). The number of nicotine-induced events with non-lateral diffusion in CTb cells was 8.1 ± 0.6, whereas for lateral diffusion the value was 2.6 ± 0.3.

Considering that the Ca^2+^ response induced by cholinergic and glutamatergic receptors agonists is altered in the trisomic CTb cells (Cárdenas et al., 1999, 2017; Rojas et al., 2008), and that nicotine did not increase the amount of exocytosis in CTb cells, we decided to induce exocytosis using the Ca^+2^ ionophore ionomycin. Upon stimulation with this compound, both CNh and CTb cells showed a significant increase in both types of exocytotic events (lateral and non-lateral diffusion; Figures 2D,E) with non-lateral diffusion events being predominant (93 ± 2 and 83 ± 3% in CNh and CTb cells, respectively). Importantly, during the 3 min recording period of ionomycin-treated cells, the number of exocytosis events with non-lateral diffusion was significantly reduced in CTb cells compared to CNh cells (*p* < 0.05), with 31 ± 3.7 (*n* = 14) events for CNh cells and 15 ± 1.9 (*n* = 14) events for CTb cells (Figure 2D). Under ionomycin stimulation, exocytotic events showing lateral diffusion accounted for only 2.1 ± 0.5 (*n* = 14) and 2.8 ± 0.5 (*n* = 14) in ionomycin-stimulated CNh and CTb cells, respectively, and were not significantly different between both cell types (Figure 2E). This suggests that only the frequency of exocytosis with no lateral diffusion is altered in CTb cells (Figure 2C).

### Decay Time Constants of pHluorin Fluorescence Signals Do Not Differ Significantly in Normal and Trisomic Cells

Due to its exquisite sensitivity to pH, pHluorin is a powerful tool to study vesicular trafficking and pH variations. When fused to a membrane protein and targeted to the lumen of acidic organelles, increase in fluorescence intensity is related to pHluorin unquenching due to increase in pH. On the other hand, dimming of pHluorin fluorescence after exocytosis could be due to vesicle retrieval and its following re-acidification, or to lateral diffusion of pHluorin after vesicle fusion (Tsuboi and Rutter, 2003; Bowser and Khakh, 2007; Jullié et al., 2014). Then, analysis of decay time constant in pHluorin fluorescence signals would give us information about the mechanisms of vesicle retrieval or endocytosis.

**Figure 3:**
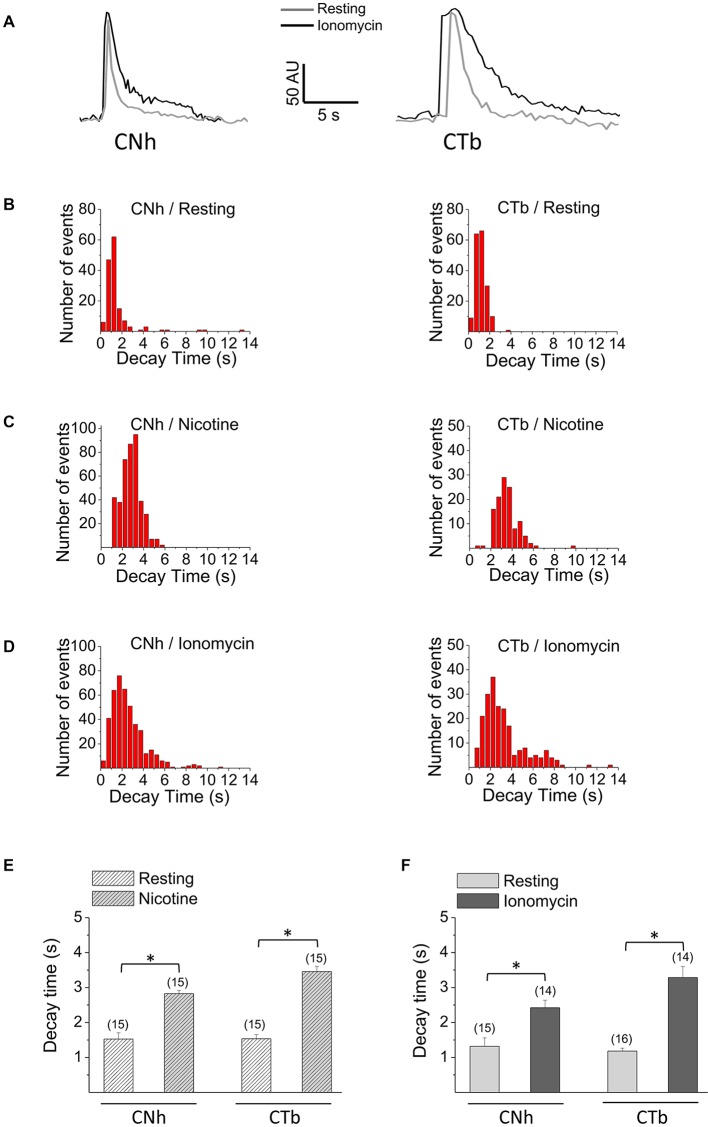
Decay kinetics of non-lateral diffusion events in CNh and CTb cells. Exocytosis was visualized using TIRF microscopy in VAChT-pHluorin expressing CNh or CTb cells in resting conditions or stimulated with 100 μM nicotine or 20 μM ionomycin. **(A)** Temporal fluorescence intensity profiles of non-lateral diffusion events in CNh or CTb cells in resting (gray lines) and ionomycin-stimulation (black lines) conditions. **(B–D)** Frequency distribution of decay times of CNh (right panels) and CTb cells (left panels) in resting conditions **(B)** or stimulated with nicotine **(C)** or ionomycin **(D)**. **(E,F)** Data are means ± SEM of decay times of the exocytotic events with non-lateral diffusion in CNh and CTb cells in resting conditions or stimulated with nicotine **(E)** or ionomycin **(F)**. Cells in resting conditions in **(E,F)** contain the vehicle. Numbers in parentheses indicate the number of cells analyzed from at least three independent cultures. *p < 0.05 (Kruskal-Wallis test, followed by Dunn’s Multiple Comparisons test as *post hoc*).

Examples of temporal fluorescence intensity profiles of non-lateral diffusion events in CNh or CTb cells are shown in Figure 3A. In resting or stimulated CNh or CTb cells, decay time constants of these pHluorin fluorescence signals fitted with a first order exponential decay (R > 0.95) and were non-parametrically distributed (Figures 3B–D). Decay time values of non-lateral diffusion events in resting CNh and CTb cells were 1.4 ± 0.2 s and 1.1 ± 0.1 s, respectively (Table 1). Given that decay times of pHluorin fluorescence signals with non-lateral diffusion became slower in the presence of a high concentration of HEPES, which slows down the reacidification of retrieved vesicles (Vardjan et al., 2007; Zhang et al., 2011) or bafilomycin A1, an inhibitor of the vesicular ATPase that also prevents the vesicle reacidification (Sankaranarayanan and Ryan, 2001; Roman-Vendrell et al., 2014), we evaluated the effects of these agents in our cell models. As shown in Table 1, both 100 mM HEPES and 100 nM bafilomycin A1 slowed decay times of non-lateral diffusion events of resting CNh and CTb cells, suggesting that changes in decay times reflected, at least in part, a re-acidification of the retrieved vesicles.

As compared to resting conditions, decay times significantly increased to 2.8 ± 0.1 s and 3.5 ± 0.1 in nicotine-stimulated CNh and CTb cells, respectively (Figure 3E; *p* < 0.05). Decay times also increased in ionomycin-stimulated cells, to 2.4 ± 0.2 s and 3.3 ± 0.3 s for CNh and CTb cells, respectively (Figure 3F; *p* < 0.05). However, decay times of CNh and CTb cells were not significantly different.

Examples of temporal fluorescence intensity profiles of lateral diffusion events in CNh or CTb cells are shown in Supplementary Figure S2A. In resting or stimulated CNh or CTb cells, decay time constants of lateral diffusion events fitted with a first order exponential decay (R > 0.95). They were non-parametrically distributed (Supplementary Figures S2B–D). For these types of events, decay times were 1.6 ± 0.2 s and 1.3 ± 0.2 s for CNh cells and CTb cells in resting conditions, respectively (Table 1). Neither HEPES nor bafilomycin A1 influences decay times of theses pHluorin fluorescence signals (Table 1). This is in agreement with previous reports, which proposed that decay time constants of lateral diffusion events depend on lateral diffusion of pHluorin after vesicle fusion (Bowser and Khakh, 2007; Malarkey and Parpura, 2011; Rao et al., 2014; Wang et al., 2017; Xu et al., 2017). As compared with the resting condition, decay times of lateral diffusion events significantly increased to 3.3 ± 0.1 s and 3.2 ± 0.2 in nicotine-stimulated CNh and CTb cells, respectively (Supplementary Figure S2E; *p* < 0.05). Stimulation with ionomycin also significantly increased decay time in CNh and CTb cells (2.7 ± 0.4 s and 2.9 ± 0.3 s, respectively) as compared with resting cells (Supplementary Figure S2F). No significant differences were found between decay times of lateral diffusion events of CNh and CTb cells under the different stimulation conditions.

### Rcan1 Knockdown Restored Ca^2+^-Dependent Exocytosis in Trisomic CTb Cells

To understand the molecular mechanisms responsible for defects in the exocytosis of the trisomic CTb cells, we focused on Rcan1 since it is overexpressed in DS patients suffering from Alzheimer’s symptoms (Ermak et al., 2001). Furthermore, its overexpression in adrenal chromaffin cells impairs exocytosis (Keating et al., 2008; Zanin et al., 2013).

**Figure 4:**
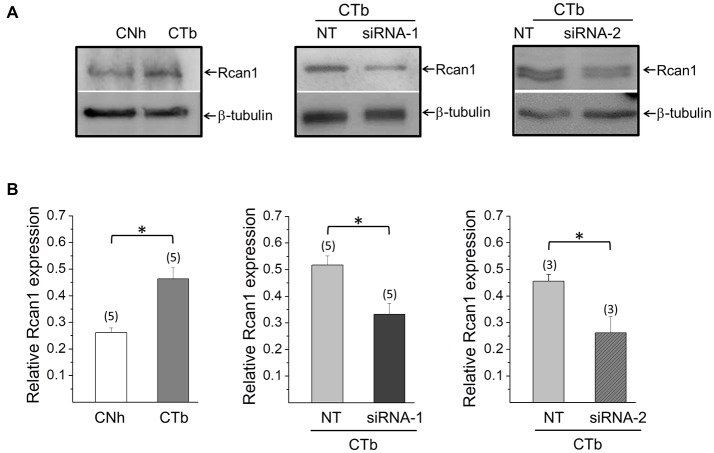
Knockdown of Rcan1 in CTb cells. Expression levels of Rcan1 in CNh and CTb cells were analyzed by western blot using a polyclonal antibody. A β-tubulin antibody was used as a loading control. **(A)** Examples of western blots of protein extracts from CNh and CTb cells, and CTb cells transfected with control non-targeting siRNA (NT) or siRNA-1 or siRNA-2 against Rcan1. Upper bands correspond to Rcan1 and bottom bands to β-tubulin. **(B)** Data are means ± SEM of normalized Rcan1 expression. Numbers in parentheses indicate the number of cell cultures analyzed. *p < 0.05 (paired *t*-test).

We first compared expression levels of Rcan1 in CTb cells with CNh cells by using Western blot assays (Figure 4A). In five independent experiments, we found that expression levels of Rcan1 is 1.8-fold higher in CTb cells as compared to CNh cells (Figure 4B). To determine whether Rcan1 overexpression is responsible for the impaired exocytosis, we knocked down Rcan1 in CTb cells using specific siRNAs and compared Rcan1 expression levels to CTb cells transfected with the NT siRNA. The expression of Rcan1 was significantly reduced 24 h after transfection of CTb cells with two different Rcan1 siRNAs, siRNA-1 and siRNA-2 (*p* < 0.05 compared with the Rcan1 expression in NT transfected CTb cells). Remarkably, expression levels of Rcan1 in that condition was restored to levels comparable to those of CNh cells (Figure 4B).

**Figure 5:**
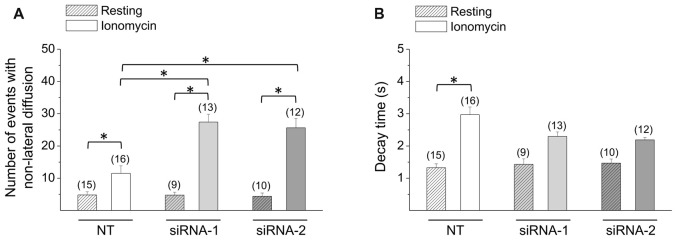
Effects of Rcan1 knockdown on the quantity and decay kinetics of non-lateral diffusion events. **(A,B)** CTb cells were transfected with VAChT-pHluorin and control non-targeting siRNA (NT), or siRNA-1 or siRNA-2 against Rcan1. Data are means ± SEM of the number **(A)** and decay times **(B)** of non-lateral diffusion events in resting or ionomycin-stimulated CTb or CNh cells. Numbers in parentheses indicate the number of cells analyzed from at least three independent cultures. *p < 0.05 (ANOVA followed by Tukey-Kramer Multiple Comparisons Test for the number of events; Kruskal-Wallis test, followed by Dunn’s Multiple Comparisons test as *post hoc* for decay times).

Next, we analyzed exocytosis events in CTb cells knocked down for Rcan1. In resting conditions, no significant alterations were observed, as the number of non-lateral diffusion events accounted for 4.8 ± 1.0 (*n* = 15) in CTb cells transfected with NT, and 4.8 ± 0.9 (*n* = 9) and 4.1 ± 1.0 (*n* = 10) in cells transfected with siRNA1 and siRNA-2, respectively. As expected, stimulation with ionomycin increased the number of exocytosis events, but interestingly the amount of exocytosis in stimulated Rcan1 knockdown CTb cells was significantly greater than that of NT-transfected CTb cells (11.5 ± 2.3 events (*n* = 16) with NT, and 27 ± 2.4 (*n* = 13) and 26 ± 2.8 (*n* = 12) events with Rcan1 siRNA1 and siRNA-2, respectively *p* < 0.05; Figure 5A). In all conditions, events with non-lateral diffusion represented over 80% of total exocytotic events. Indeed, 81 ± 6% and 88 ± 3% for NT siRNA-transfected cells in resting and stimulation conditions, and 97 ± 3% and 90 ± 2% for Rcan1 knockdown CTb cells with siRNA-1 in resting and stimulation conditions, and 81 ± 5% and 89 ± 1% for Rcan1 knockdown CTb cells with siRNA-2 in resting and stimulation conditions, respectively.

Like non-transfected cells, decay times of transfected non-lateral diffusion decayed with first-order kinetics (*R* > 0.95). In resting conditions, decay time constants were 1.3 ± 0.1 s, 1.4 ± 0.2 s and 1.5 ± 0.1 s for CTb cells transfected with NT, siRNA-1 or siRNA-2, respectively. As shown in Figure 5B, decay time significantly increased upon stimulation with ionomycin in CTb cells transfected with NT (*p* < 0.05 compared with resting CTb cells in each condition), however it did not in cells transfected with siRNA-1 or siRNA-2. Rcan1 knockdown with siRNA-1 or siRNA-2 did not significantly affect decay time constants as compared with NT-siRNA transfected cells.

Regarding the number of events with lateral diffusion, the small amount of this type of event observed did not allow us to perform statistical analyses. Indeed, CTb cells transfected with NT or Rcan1 siRNAs exhibited less than one event in resting conditions. In ionomycin-stimulated condition, they amounted to 1.0 ± 0.3, 2.8 ± 0.7 and 3.2 ± 0.4 events in CTb cells transfected with NT, siRNA-1 or siRNA-2, respectively.

## Discussion

Cholinergic transmission plays a crucial role in memory and learning (Thiel, 2003; Voss et al., 2012; Handjaras et al., 2013). In Ts65Dn mice, an animal model of DS, a reduced hippocampal release of acetylcholine during memory performances correlates with poor performance in tasks designed to evaluate working memory (Chang and Gold, 2008). Diminished acetylcholine release has also been observed in primary cultures of neurons from Ts16 mice brains (Fiedler et al., 1994), as well as in the trisomic CTb cell line (Allen et al., 2000). In the present work, we have characterized the modes by which VAChT-pHluorin is released in the trisomic CTb cells. Our major findings indicate that i) the amount of Ca^2+^ exocytosis is reduced in CTb cells; and ii) reducing expression levels of Rcan1 in CTb cells at levels comparable to CNh cells restore Ca^2+^-dependent exocytosis.

### Do Non-lateral and Lateral Diffusion Events Reflect Different Modes of Exo/Endocytosis?

Using vesicle associated pHluorin as a reporter, TIRF microscopy allows single exocytosis event analysis by detecting bright flashes of fluorescence, and to extract the rate constant from fluorescence intensity variation (Poulter et al., 2015). In CNh and CTb cells maintained in resting or stimulated conditions, over 80% of the fluorescence flashes showed non-lateral diffusion of VAChT-pHluorin. This restricted diffusion pattern of pHluorin-tagged proteins has been attributed to the retrieval of vesicle components at fusion sites and is reminiscent of the kiss-and-run mechanism of exo/endocytosis (Jullié et al., 2014; Xu et al., 2017). This mechanism relies on the transient opening of the fusion pore, a narrow channel formed during exocytosis (Lindau and Alvarez de Toledo, 2003; Mosharov and Sulzer, 2005), which in turn restricts the release of transmitters (Albillos et al., 1997; Alés et al., 1999; Alabi and Tsien, 2013). The kiss-and-run mechanism has been described in hippocampal synapses (Stevens and Williams, 2000; Gandhi and Stevens, 2003; Harata et al., 2006; Zhang et al., 2009), calyx of Held nerve terminal (He et al., 2006) and dorsal root ganglion neurons (Wang et al., 2017).

On the other hand, lateral diffusion events have been associated with full fusion exocytosis (Taraska et al., 2003; Tsuboi and Rutter, 2003; Jullié et al., 2014). In this mode of exocytosis, the entire vesicle membrane merges with the plasma membrane, and consequently vesicle proteins spread out by lateral diffusion through the plasma membrane (Taraska et al., 2003). However, the non-lateral and lateral diffusion events observed in the CNh and CTb cells could not necessarily correlate with these two modes of exocytosis, since, on one hand, selected vesicle membrane proteins can be released in some forms of kiss-and-run (Tsuboi and Rutter, 2003; Tsuboi et al., 2004), and, on the other hand, some vesicle membrane proteins do not spread after full fusion, and remain clustered at fusion sites until endocytosis begins (Opazo et al., 2010; Ceridono et al., 2011). However, it is important to consider that in the latter case, the recapture of the vesicle proteins takes minutes, whereas, in our case VAChT-pHluorin fluorescence decayed in seconds, and with values comparable to reported kiss-and-run events (Tsuboi and Rutter, 2003; Tsuboi et al., 2004; Bowser and Khakh, 2007).

### Reduced Amount of Exocytosis in the Trisomic CTb Cells

In contrast to a recent study using the Ts65Dn mouse model (Marland et al., 2016), we found that in response to ionomycin, CTb cells showed a decrease in exocytosis frequency compared to control CNh cells (Figure 2B). Several reasons may explain this discrepancy. The first one might be the difference of genetic background between the Ts16 and Ts65Dn mice used in both studies. Ts65Dn mouse carries a segmental trisomy of the chromosomes 16 and 17 (Reeves et al., 1995), whereas chromosome 16 is completely triplicated in the Ts16 mouse (Reeves et al., 1987; Coyle et al., 1991). Nevertheless, both mouse models carry most of the gene complement of the Down syndrome critical region, including RCAN1, APP, SOD1 and DYRK1A (Reeves et al., 1995; Gardiner et al., 2003; Lange et al., 2004; Duchon et al., 2011). The second explanation relies on image acquisition frequency. Indeed, whereas we acquired 1 image every 300 ms (3.3 Hz), Marland et al. (2016) recorded 1 image every 4 s (0.25 Hz). According to the mean fluorescence decay time measured in our study (less than 4 s), it is likely that, at 0.25 Hz, some events were missed and hence did not yield a full comprehensive view of exocytotic events. Nonetheless, our results are in line with neurotransmitter release defects observed in models of DS (Fiedler et al., 1994; Chang and Gold, 2008), suggesting that regulated-exocytosis is indeed altered in CTb cells.

### The Knockdown of Rcan1 Increases the Extent of Exocytosis in CTb Cells

Among the genes amplified in DS cells, we focused our attention on Rcan1 since its overexpression impairs the extent of exocytosis in adrenal chromaffin cells (Keating et al., 2008; Zanin et al., 2013). We confirmed that Rcan1 is overexpressed in CTb cells compared to CNh cells (Figure 4), and to address its contribution to the regulation of exo/endocytosis, we reduced its expression by transfecting specific Rcan1 siRNAs. In this condition, RCan1 abundance reached levels comparable to those observed in CNh cells. Interestingly, although Rcan1 knockdown in CTb cells did not affect spontaneous exocytosis, it increased the number of non-lateral diffusion events induced by ionomycin, reaching values similar to those of CNh cells. Therefore, as in chromaffin cells, our study indicates that Rcan1 is involved in the control of regulated exocytosis.

The effects of the Rcan1 overexpression on exocytosis in chromaffin cells seem to be a consequence of the chronic inhibition of calcineurin. Indeed, chronic exposure of chromaffin cells to calcineurin inhibitors reduced the total amount of exocytosis and impaired vesicle recycling (Zanin et al., 2013). Among the calcineurin substrates involved in vesicle recycling are dynamin, amphiphysin and synaptojanin (Cousin and Robinson, 2001). Of particular interest is dynamin, which in addition to its role in endocytosis, regulates exocytosis and vesicle recycling (González-Jamett et al., 2010, 2013; Moya-Díaz et al., 2016). Rcan1 also regulates, via calcineurin, the actin cytoskeleton dynamics by regulating cofilin phosphorylation (Wang et al., 2016), a protein that disassembles actin filaments (Maciver and Hussey, 2002; Pavlov et al., 2007; Pfaendtner et al., 2010). It is known that Rcan1 overexpression reduces the levels of active cofilin (Wang et al., 2016), and cofilin knockdown decreases G-actin/F-actin ratio (Hotulainen et al., 2005). Since actin plays critical roles at different stages of exocytosis (Porat-Shliom et al., 2013; Olivares et al., 2014) Rcan1 overexpression could have affected exocytosis in CTb cells by impairing actin remodeling. Interestingly, CTb cells exhibit increased F-actin/G-actin ratio when compared with CNh cells (Pérez-Núñez et al., 2016) and it has been observed that a reduced F-actin disassembly negatively impacts exocytosis (Meunier and Gutiérrez, 2016).

## Conclusion

Hundreds of genes are overexpressed in DS (Hattori et al., 2000), which have a variable contribution to the phenotypes associated with the trisomic condition. Although the molecular mechanisms remain to be addressed, we found that the overexpression of Rcan1 contributes to the reduced exocytosis function in the trisomic condition. Rcan1 knockdown is apparently sufficient to restore such impaired exocytosis, even when other genes of the DS critical region, such as the amyloid precursor protein and the DS cell adhesion molecule, are also overexpressed (Opazo et al., 2006; Rojas et al., 2008; Pérez-Núñez et al., 2016).

## Author Contributions

JV-N performed experiments and statistical analysis. AM designed and interpreted results. SO interpreted results and critically revised the manuscript. XB-M performed experiments. AG-J performed experiments and critically revised the manuscript. SB designed constructs, interpreted results and critically revised the manuscript. PC conceived the study, interpreted results and critically revised the manuscript. AC conceived the study, designed experiments, interpreted results and drafted the manuscript. All authors read and approved the final manuscript.

## Conflict of Interest Statement

PC holds patent protection for the CNh and CTb cell lines.

## Abbreviations

DS: Down syndrome
DSCR1: Down syndrome critical region 1
NT: non-targeting siRNA
RCAN1: regulator of calcineurin 1
Ts16: trisomy 16
TIRFM: total internal reflection fluorescence microscopy
VAChT-pHluorin: vesicular acetylcholine transporter fused to the pH-sensitive green fluorescent protein.
