# Supplementary material for: RCAN1 Knockdown Reverts Defects in the Number of Calcium-Induced Exocytotic Events in a Cellular Model of Down Syndrome
Source: Front Cell Neurosci. 2018 Jul 6;12:189. doi: 10.3389/fncel.2018.00189 (PMC6043644; doi:10.3389/fncel.2018.00189)
Supplement: Supplementary file 1 [file Image_1.pdf]

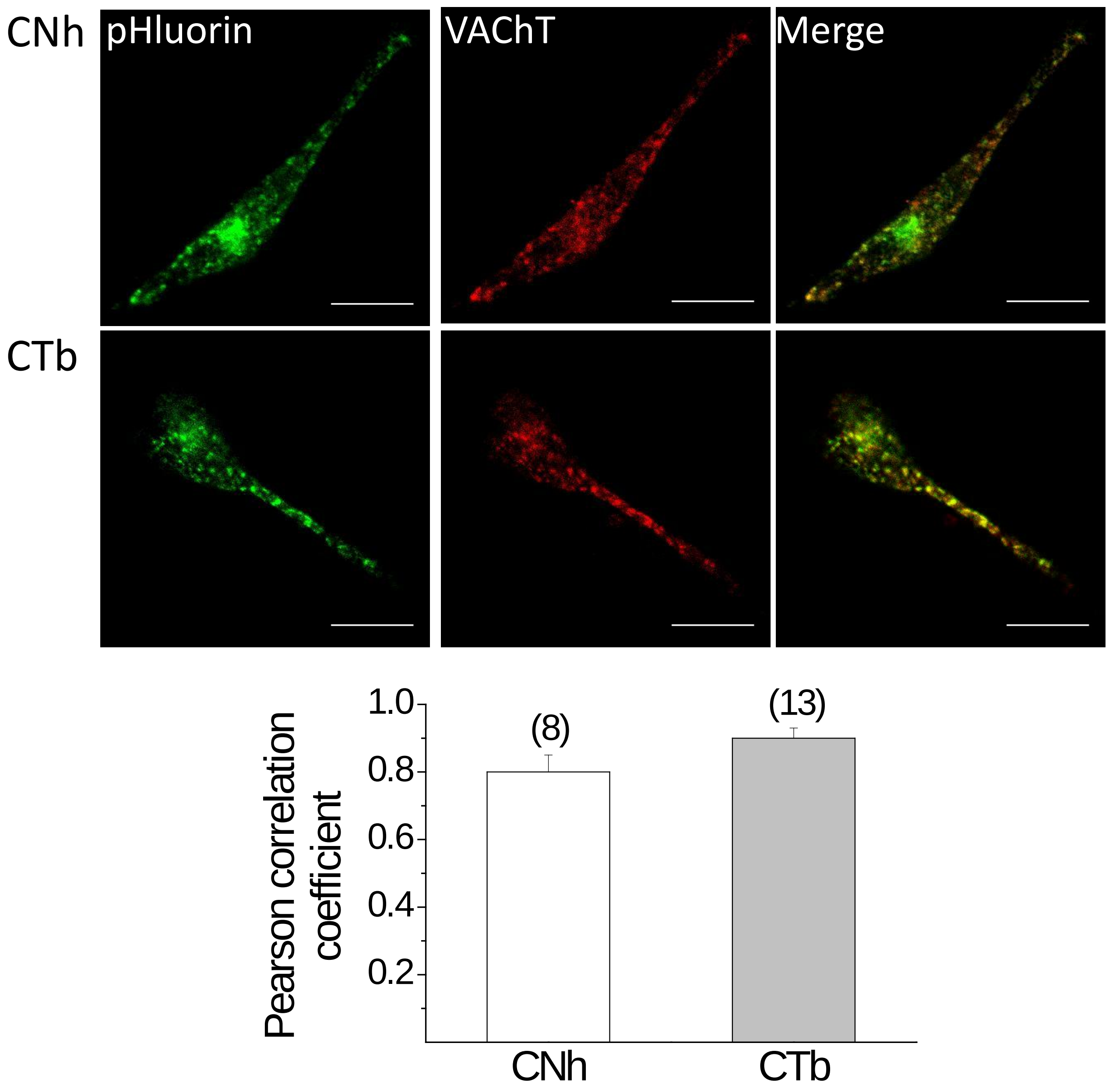

Figure S1: *VAChT-pHluorin colocalizes with endogenous VAChT-enriched structures in CNh and CTb cells.* CNh and CTb cells cultured in 25 mm coverslips were transfected with VAChT-pHluorin and 24h later were fixed, stained with an antibody directed against the cholinergic vesicle marker VAChT and visualized by confocal microscopy. (A) Representative images of CNh (upper panels) and CTb cells (bottom panels) expressing VAChT-pHluorin (green) and VAChT-positive vesicles (red). Right panels show merged images. Scale bar =10 µm. (B) Graph shows the mean Pearson correlation coefficient for CNh (light gray bar) and CTb (dark gray bar) cells. Data are means ± SEM. Numbers in parentheses indicate the number of cells analyzed from at least three independent cultures.
