# Supplementary material for: RCAN1 Knockdown Reverts Defects in the Number of Calcium-Induced Exocytotic Events in a Cellular Model of Down Syndrome
Source: Front Cell Neurosci. 2018 Jul 6;12:189. doi: 10.3389/fncel.2018.00189 (PMC6043644; doi:10.3389/fncel.2018.00189)
Supplement: Supplementary file 2 [file Image_2.PDF]

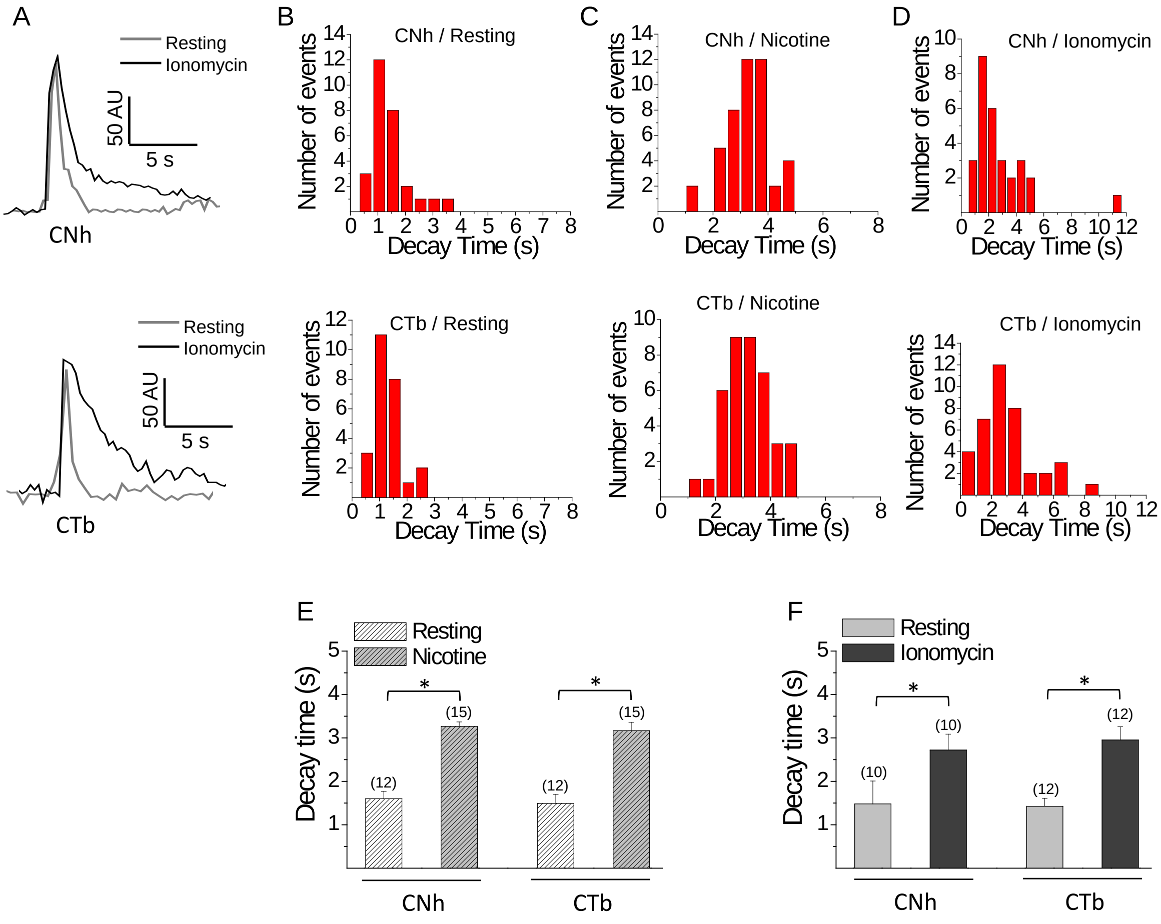

Figure S2: *Decay kinetics of exocytotic events with lateral diffusion in CNh and CTb cells*. Exocytosis was visualized using TIRF microscopy in VAChT-pHluorin expressing CNh or CTb cells in resting conditions or stimulated with 100 µM nicotine or 20 µM ionomycin. (**A**) Temporal fluorescence intensity profiles of lateral diffusion events in CNh (upper panel) or CTb cells (lower panel) in resting (grey lines) and ionomycin-stimulation (black lines) conditions. (**B-D**) Frequency distribution of decay times of CNh (upper panels) and CTb cells (lower panels) in resting conditions (B) or stimulated with nicotine (C) or ionomycin (D). (**E-F**) Data are means ± SEM of decay times of the exocytotic events with non-lateral diffusion in CNh and CTb cells in resting conditions or stimulated with nicotine (E) or ionomycin (F). Cells in resting conditions in (E) and (F) contain the vehicle (0.002% ethanol in E and 1% DMSO in F). Numbers in parentheses indicate the number of cells analyzed from at least three independent cultures. *$p < 0.05$ (Kruskal-Wallis test, followed by Dunn's Multiple Comparisons test as posthoc).
